# Endovascular treatment of acute basilar artery occlusion: A systematic review and meta‐analysis of first‐line stent retriever versus direct aspiration

**DOI:** 10.1002/brb3.3141

**Published:** 2023-07-11

**Authors:** Juan Zhang, Yongbin Wang, Yanmei Ju, Hongxin Jiang

**Affiliations:** ^1^ Department of Neurology Gucheng Hospital in Hebei Province Hengshui China; ^2^ Department of Neurosurgery Gucheng Hospital in Hebei Province Hengshui China; ^3^ Department of Gynaecology and Obstetrics Gucheng Hospital in Hebei Province Hengshui China; ^4^ Department of Radiology Gucheng Hospital in Hebei Province Hengshui China

**Keywords:** acute basilar artery occlusion, aspiration, endovascular treatment, meta‐analysis, stent retriever

## Abstract

**Background:**

The best choice between first‐line aspiration and stent retriever for acute basilar artery occlusion remains controversial. This study aims to perform a systematic review and meta‐analysis comparing the stent retriever and direct aspiration about reported recanalization rates and periprocedural complications.

**Method:**

PubMed, Embase, Web of Science, Cochrane, and Clinical Trials were searched for the studies evaluating the efficacy and safety of first‐line aspiration versus stent retriever for acute basilar artery occlusion. A standard software program (Stata Corporation) was used for end‐point analyses. Statistical significance was defined as a *p*‐value less than .05.

**Results:**

A total of 11 studies were involved in the current study, including 1014 patients. Regarding postoperative recanalization, the pooled analysis identified a significant difference in successful recanalization (odds ratio [OR] = 1.642; 95% confidence interval (95% CI): 1.099–2.453; *p* = .015) and complete recanalization (OR = 3.525; 95% CI: 1.306–2.872; *p* = .001) between the two groups in favor of the first‐line aspiration. Concerning the complications, the first‐line aspiration could achieve a lower rate of total complication (OR = .359; 95% CI: .229–.563; *p* < .001) and hemorrhagic complication (OR = .446, 95% CI: .259–.769; *p* = .004) than stent retriever. No significant difference was observed in postoperative mortality (OR = .966; *p* = .880), subarachnoid hematoma (OR = .171; *p* = .094), and parenchymal hematoma (OR = .799; *p* = .720). In addition, the pooled results revealed a significant difference in procedure duration between the two groups in favor of aspiration (WMD = −27.630, 95% CI: −50.958 to −4.302; *p* = .020). However, there was no significant difference in favorable outcome (OR = 1.149; *p* = .352) and rescue therapy (OR = 1.440; *p* = .409) between the two groups.

**Conclusion:**

Given that the first‐line aspiration was associated with a higher rate of postoperative recanalization, a lower risk of postoperative complication, and a faster duration of the procedure, these findings support the aspiration may be more secure than a stent retriever.

## Graphical abstract

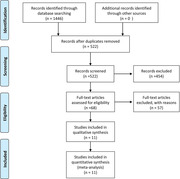

## 1 INTRODUCTION

Acute basilar artery occlusion, a type of posterior circulation stroke, accounts for approximately 1%–4% of all stroke cases and has a high mortality risk (Demel & Broderick, 2015). Unlike hemispheric ischemia, which usually has a fast onset of localized symptoms, basilar artery occlusion syndromes might mimic other non‐stroke diseases, causing neurological diagnosis to be delayed (Sarraj et al., 2015). The most frequent causes include atherosclerotic occlusion caused by local thrombosis due to severe stenosis and embolic occlusions, poor collateral circulation, and inadequate cerebral tissue perfusion (Zhao et al., 2023). It is crucial to restore blood perfusion by opening the occluded vessels on time. Several treatment approaches are currently available: thrombolytic therapy including intravenous thrombolysis and intra‐arterial thrombolysis, mechanical thrombectomy, or combinations of these (Wyszomirski et al., 2017; Yu & Higashida, 2022).

Intravenous thrombolysis refers to the systemic administration of recombinant tissue plasminogen activator. Theoretically, intravenous thrombolysis can initiate faster in most hospitals and the thrombolytic agent could reach the clot from both distal and proximal aspects. Intra‐arterial thrombolysis allows for a higher local concentration of fibrinolytic while maintaining a lower systemic concentration (Lindsberg et al., 2012). In randomized trials, the effectiveness of endovascular treatment for basilar artery occlusions has been established recently, demonstrating its superiority over medical treatment alone (Jovin et al., 2022; Malik et al., 2022; Tao et al., 2022). Mechanical thrombectomies, such as aspiration thrombectomy, angioplasty with stenting, and stent retriever thrombectomy, have been tested with variable recanalization and clinical outcomes (Marmagkiolis et al., 2015). Stent retrievers have risen to prominence in the last few years. The majority of stent retrievers utilized for mechanical thrombectomy are self‐expanding tubular stent‐like devices with sophisticated mesh designs that allow better clot integration and removal (Piasecki et al., 2022). This procedure recanalizes the vessel, allowing high rates of prompt flow restoration but leading to in‐stent restenosis and in‐stent thrombosis.

Catheter technology advancements have provided flexible and large‐bore catheters that can safely navigate intracerebral circulation. A direct aspiration first‐pass technique has shown equivalent recanalization rates and neurological recovery to stent retriever thrombectomy when used either alone or in conjunction with stent retrievers (Nogueira et al., 2018; Turk et al., 2015). Direct aspiration is thought to facilitate recanalization and reduce the expense of endovascular interventions (Turk et al., 2014). A direct aspiration first‐pass technique is a fast, simple, efficient, and safe strategy to achieve revascularization in patients. The best choice for basilar artery occlusion remains controversial. This study aims to perform a systematic review and meta‐analysis comparing the stent retriever and direct aspiration concerning reported recanalization rates and periprocedural complications.

## 2 METHODS

### 2.1 Literature search strategy

This study was conducted according to the Preferred Reported Items for Systematic Reviews and Meta‐Analyses (PRISMA) guideline (McInnes et al., 2018; Zaks et al., 2023). Two independent reviewers systematically obtained publications from four electronic databases including PubMed, Embase, Web of Science, Cochrane, and Clinical Trials to explore the safety and efficacy of first‐line aspiration versus stent retrievers in treating patients with acute basilar artery occlusion from the inception of electronic databases to March 2023. The search strategy adopted both keywords and the MeSH term searches about “stent” and “basilar artery occlusion,” which were also in combination with Boolean logic. In addition, other potentially relevant studies were manually discovered from references of eligible research or reviews on this subject.

### 2.2 Inclusion and exclusion criteria

All retrieved publications were independently extracted by two investigators. Studies were included in this meta‐analysis if they fulfilled the following predefined criteria: (1) randomized controlled trials or observational cohort studies in English; (2) comparison of the safety of aspiration and stent retriever in treating basilar artery occlusion; and (3) one or more outcomes were reported.

The exclusion criteria were listed as follows: (1) animal study; (2) the data in the study could not be extracted; (3) studies that were not comparative, such as case reports, reviews, conference or commentary articles, letters, surveys, or satisfaction studies; (4) studies only equipped with a single‐arm design; and (5) studies not meeting inclusion criteria.

### 2.3 Data extraction and outcome measures

Two authors independently extracted baseline characteristics, primary outcomes, and secondary outcomes from each eligible included study by using a form prepared in advance. Baseline data included first author, year of publication, study period, sample size, sex, age, and initial NIHSS score. Any question was discussed and resolved until a final decision. The primary end point of this meta‐analysis included successful recanalization (defined as Thrombolysis in Cerebral Ischemia 2b or 3), complete recanalization (defined as Thrombolysis in Cerebral Ischemia 3), total complication, hemorrhagic complication, mortality, subarachnoid hematoma, parenchymal hematoma, a favorable outcome, rescue therapy, and procedure duration. Rescue therapy encompasses a range of interventions aimed at overcoming the potential failure of initial endovascular thrombectomy. These interventions may include switching to an alternative endovascular thrombectomy technique, employing angioplasty, utilizing adjuvant stenting, administering thrombolytics, and implementing antiplatelet medications.

### 2.4 Quality assessment

The quality assessment of each included study was conducted by two independent authors. The Newcastle–Ottawa Scale was used to evaluate the quality of observational cohort studies included in this study, which gives a score out of a possible total of nine stars (Guo et al., 2023). Any disagreement during the quality assessment procedure was resolved by consulting with the corresponding author.

### 2.5 Statistical analysis

This study adopted a standard software program (Stata Corporation) for end‐point analyses. To assess the heterogeneity among the studies, we utilized the Higgins *I*‐square (*I* ^2^), a metric that represents the proportion of variability attributable to differences between studies. When the *I* ^2^ value was below 50%, it indicated homogeneity in the end‐point measure. In such cases, we conducted a meta‐analysis using a fixed‐effect model, following the guidelines outlined in the *Cochrane Handbook for Systematic Reviews of Interventions*. Conversely, if the *I* ^2^ value exceeded 50%, we employed a random‐effects model for the analysis. Meanwhile, as the included studies were not randomized controlled trials providing relative risk ratios, the outcomes of discontinuous outcomes (successful recanalization, complete recanalization, total complication, hemorrhagic complication, mortality, subarachnoid hematoma, parenchymal hematoma, favorable outcome, and rescue therapy) were expressed as odds ratios (ORs) with 95% confidence intervals (CIs). Procedure duration, the continuous end point, was represented as a weighted mean difference (WMD) with 95% CIs. Statistical significance was defined as a *p*‐value less than .05.

## 3 RESULTS

### 3.1 Search results

**FIGURE 1:**
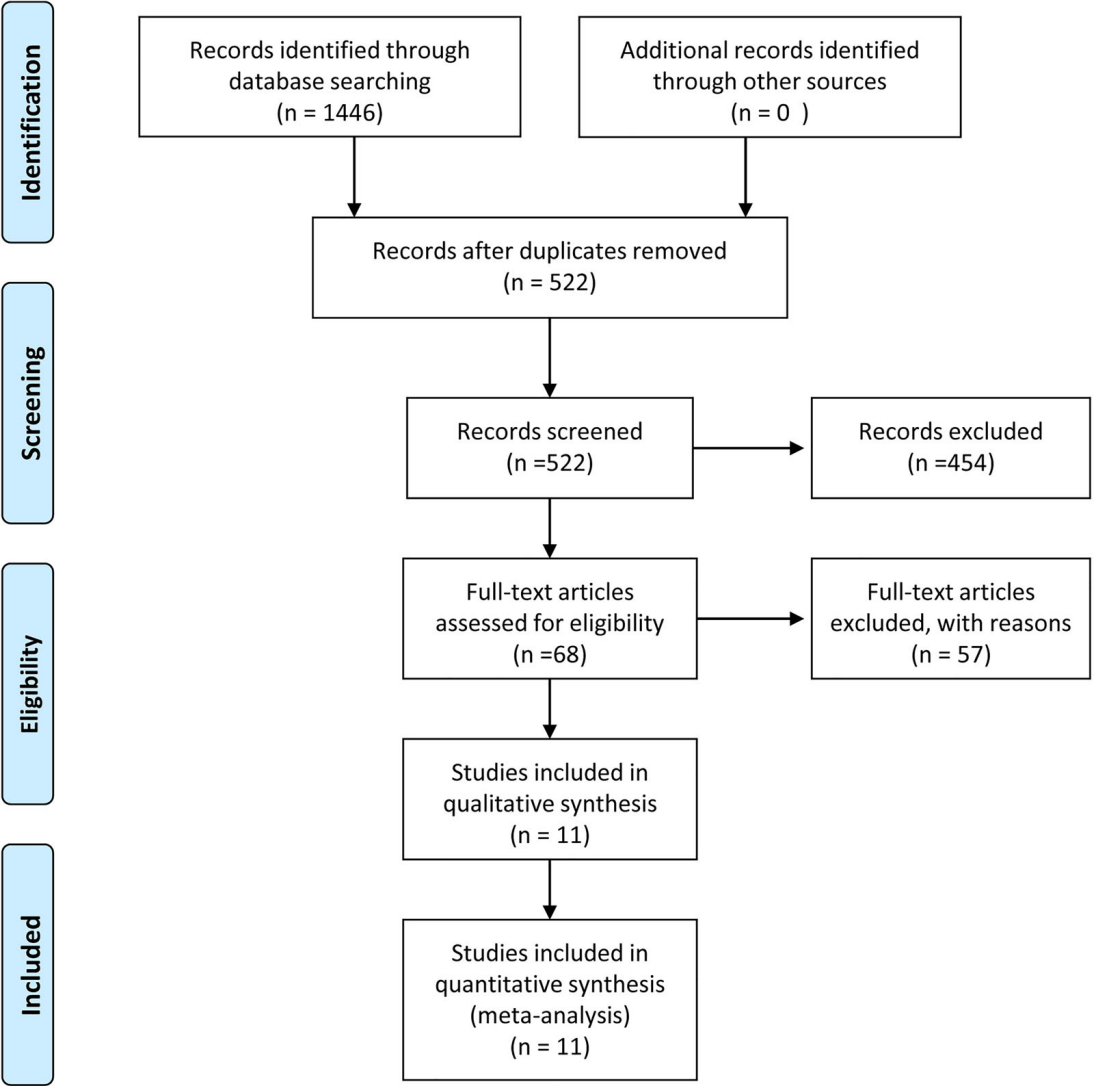
The specific screening study process.

According to the constructed searching strategy, 1446 research papers were found in our systematic search across four databases (PubMed, Embase, Web of Science, Cochrane, and Clinical Trials). Figure 1 illustrates the study selection procedure. In brief, a total of 924 duplicated publications were removed in the process of importing the searching articles to the Endnote software. Of 522 articles, 454 were excluded for various reasons (case reports, case series, reviews, letters, meta‐analysis, and irrelevant articles) via screening the title and abstract. After that, the full texts of 68 papers were read two times and scrutinized for data integrity and adherence to the inclusion and exclusion criteria. Finally, we enrolled 11 clinical studies in our present systematic review and meta‐analysis (Abdelrady et al., 2023; Baik et al., 2021; Choi et al., 2020; Gerber et al., 2017; Giorgianni et al., 2018; Gory et al., 2018; Kaneko et al., 2021; Kang et al., 2018; Li et al., 2018; Mokin et al., 2016; Son et al., 2016).

### 3.2 Study characteristics and quality assessment

Demographic characteristics and details regarding the study design of the involved studies are summarized in Table 1. The period of publication and data collection of the studies included in the meta‐analysis was from 2014 to 2022 and 2010 to 2019. This study involved 1014 participants (410 were treated with first‐line aspiration and 604 with stent retriever), and the sample size varied from 31 to 212. The studies were from Germany, Japan, Korea, Italy, France, Germany, and USA. Two reviewers used the Newcastle–Ottawa Scale to determine the quality of the included prospective and retrospective studies. When there were conflicts, an additional authoritative author was brought in to mediate the issue until a consensus was reached. Table 2 displays the characteristics and comprehensive quality evaluations of the 11 included studies.

**TABLE 1:** Baseline characteristics of the included studies.

			Patients (number)		Participant age (year)		Male (percentage)		Initial NIHSS	
Reference	Published year	Collection period	Aspiration	SR	Aspiration	SR	Aspiration	SR	Aspiration	SR
Abdelrady et al. (2023)	2022	2015–2019	33	35	67.5 ± 11	65.97 ± 13.7	58	57	14 (8–19.5)	18 (12–40)
Baik et al. (2021)	2021	2013–2019	43	118	Not report	Not report	Not report	Not report	Not report	Not report
Choi et al. (2020)	2020	2016–2019	34	16	65.25 (51–84)	69.38 (18–89)	62.5	62.5	13.5–22.25	13.25–26.75
Kaneko et al. (2021)	2021	2015–2019	38	21	77 (68–84)	77 (68–84)	64.4	64.4	24 (13–30)	24 (13–30)
Giorgianni et al. (2018)	2018	2010–2015	27	60	68 (57–76)	68 (57–76)	63.7	63.7	17	17
Gory et al. (2018)	2018	2010–2016	46	54	61 (53–71)	67 (53–78)	58.7	63	6 (6–9)	8 (6–8)
Kang et al. (2018)	2018	2010–2016	67	145	71 (64–78)	71 (64–78)	64–78	64–78	17 (10–24)	17 (10–24)
Mokin et al. (2016)	2016	2012–2015	42	58	63.5 ± 14.2	63.5 ± 14.2	67	67	19.2 ± 8.2	19.2 ± 8.2
Son et al. (2016)	2014	2011–2013	18	13	66.4 ± 11.4	69.8 ± 10.4	77.8	53.8	21.3 ± 9.7	27.3 ± 11
Gerber et al. (2017)	2017	2013–2016	20	13	57.4–68.2	54.1–72.3	70	62	18 (4–32)	25 (16–33)
Li et al. (2018)	2017	2012–2016	40	71	Not report	Not report	Not report	Not report	Not report	Not report

Abbreviations: NIHSS, national institutes of stroke scale; SR, stent retriever.

**TABLE 2:** Quality assessment scores of the included studies.

		Newcastle–Ottawa Scale (NOS)			
Study (year)	Design	Selection	Comparability	Exposure	Total scores
Abdelrady et al. (2023)	Retrospective cohort study	3	1	3	7
Baik et al. (2021)	Prospective cohort study	3	2	3	8
Choi et al. (2020)	Retrospective cohort study	3	2	3	8
Kaneko et al. (2021)	Retrospective cohort study	3	2	3	8
Giorgianni et al. (2018)	Retrospective cohort study	3	2	2	7
Gory et al. (2018)	Prospective cohort study	3	2	3	8
Kang et al. (2018)	Prospective cohort study	3	2	3	8
Mokin et al. (2016)	Retrospective cohort study	3	1	3	7
Son et al. (2016)	Retrospective cohort study	3	2	2	7
Gerber et al. (2017)	Retrospective cohort study	3	1	3	7
Li et al. (2018)	Retrospective cohort study	3	2	3	8

### 3.3 Outcomes of meta‐analysis

#### 3.3.1 Postoperative recanalization

**FIGURE 2:**
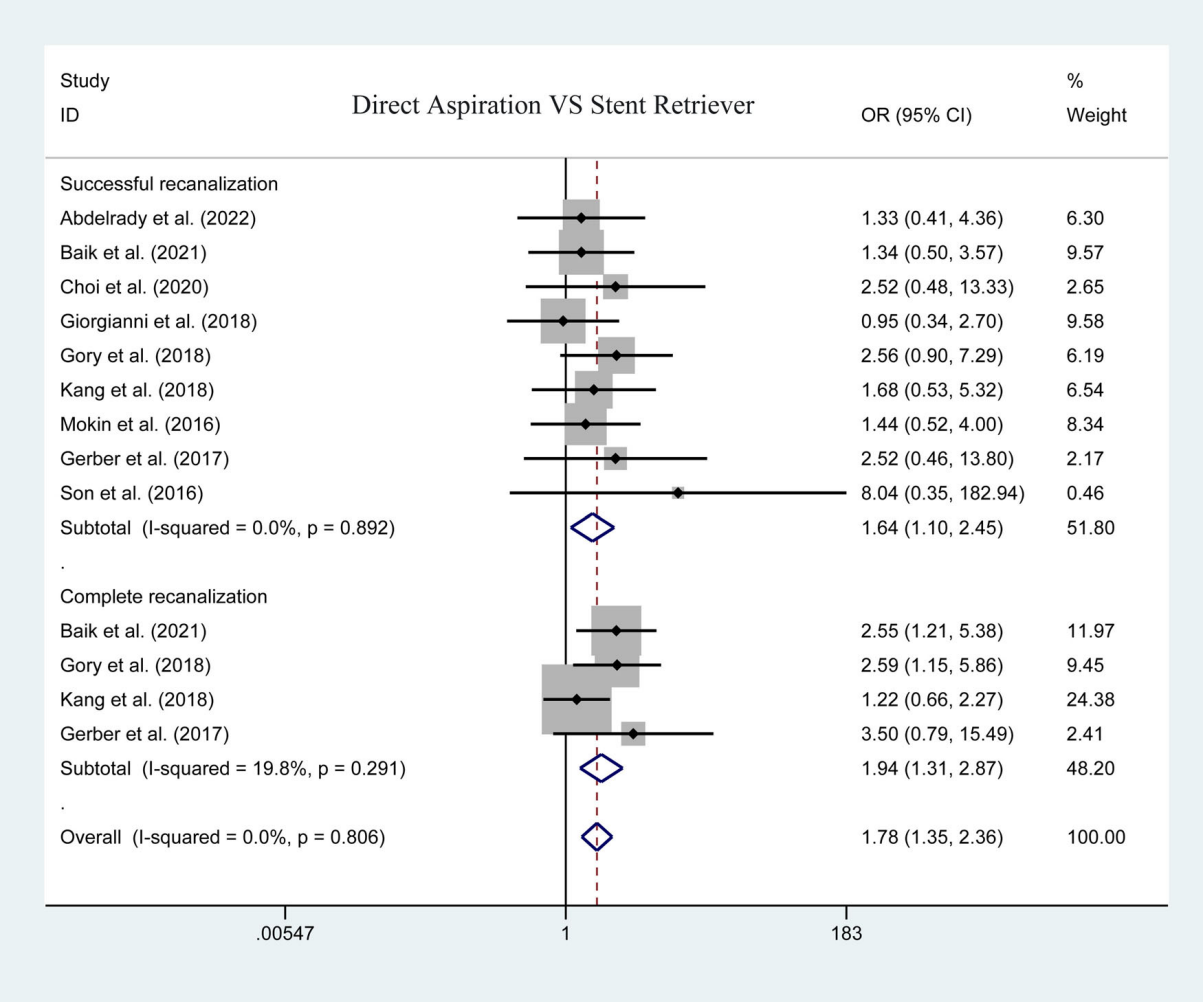
Forest plot for assessing postoperative successful recanalization and complete recanalization.

Figure 2 illustrates the pooled data of the successful recanalization. In 9 studies, 312 and 530 patients were enrolled in first‐line aspiration and stent retriever, respectively. The pooled analysis identified a significant difference between the two groups (heterogeneity *p* = .892; *I* ^2^ = .0%; OR = 1.642; 95% CI: 1.099–2.453; *p* = .015; Figure 2) in favor of the first‐line aspiration. Further subgroup analysis was conducted in complete recanalization, the rate of complete recanalization in the first‐line aspiration was significantly higher than that in the stent retriever group (heterogeneity *p* = .291; *I* ^2^ = 19.8%; OR = 1.936; 95% CI: 1.306–2.872; *p* = .001; Figure 2).

#### 3.3.2 Postoperative surgical complications and specific complications

**FIGURE 3:**
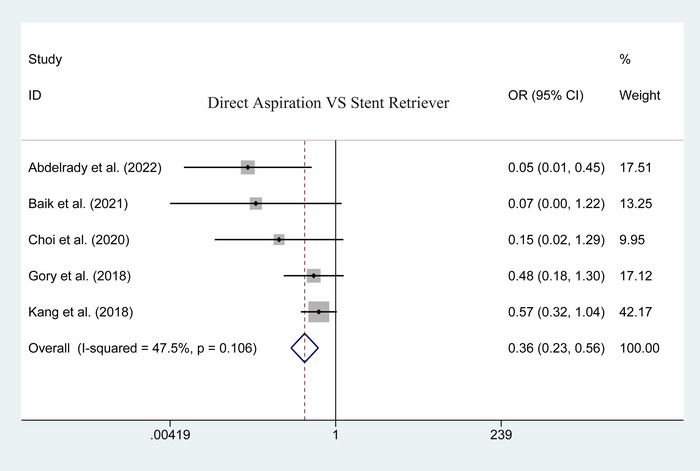
Forest plot for assessing postoperative total complications.

**FIGURE 4:**
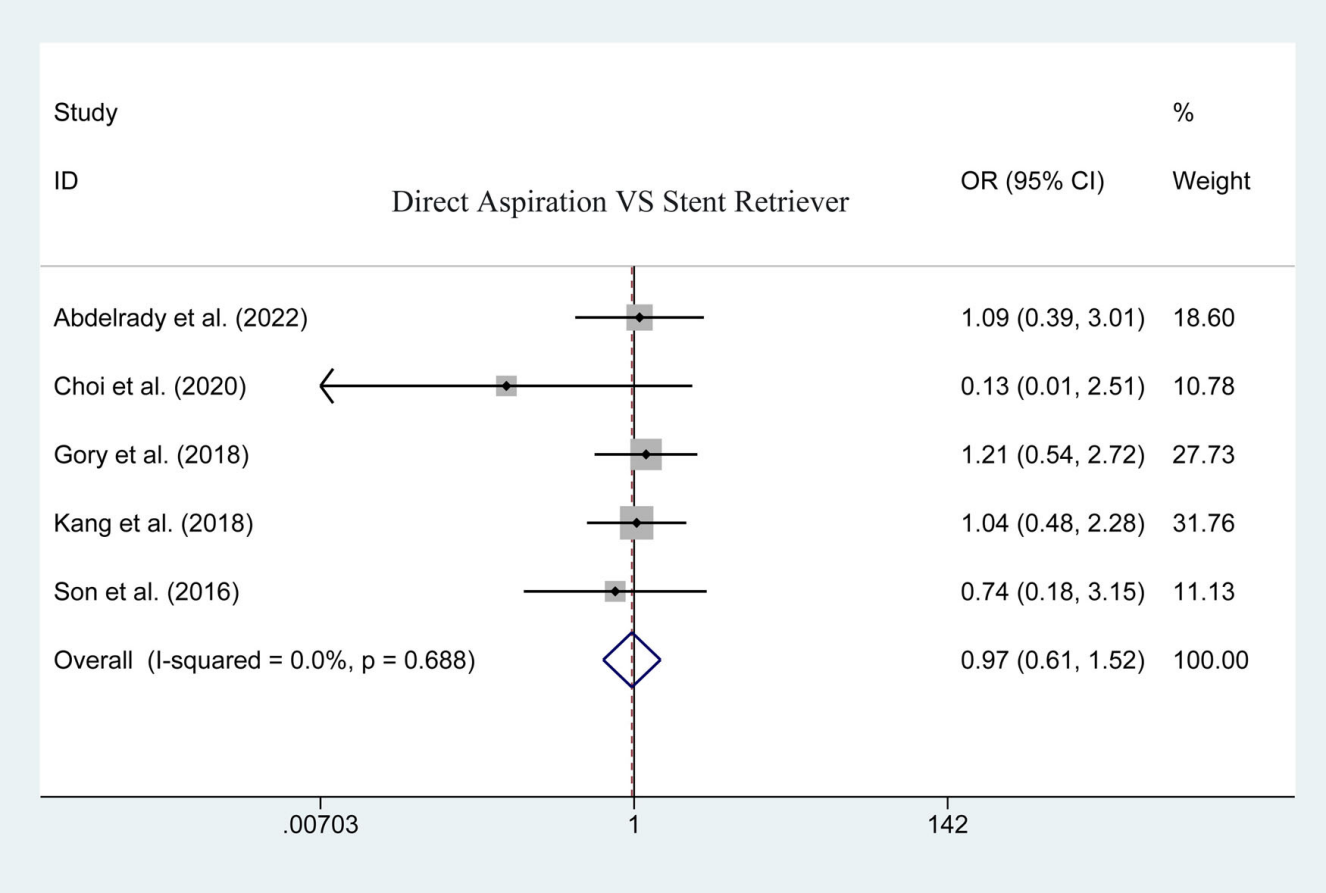
Forest plot for assessing postoperative mortality.

**FIGURE 5:**
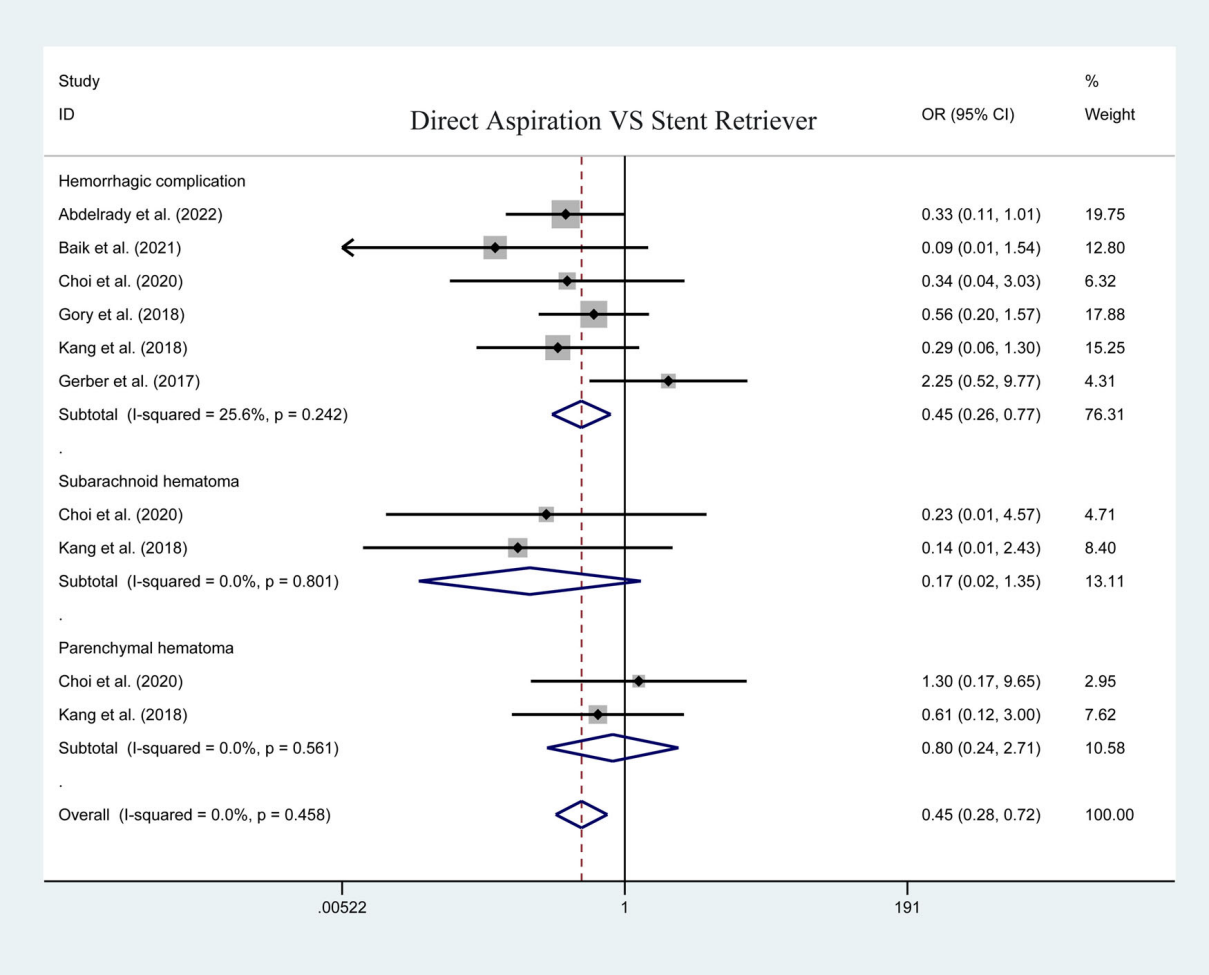
Forest plot for assessing postoperative hematoma.

Five studies have been reported on the postoperative total complication between the two groups. No significant heterogeneity in the statistical results of the pooled analysis (*I* ^2^ = 47.5%; heterogeneity *p* = .106). The result using the fixed‐effect model showed that the first‐line aspiration could achieve a lower rate of postoperative total complication than the stent retriever (OR = .359; 95% CI: .229–.563; *p* < .001; Figure 3). Upon the specific complications, no significant difference was detected in postoperative mortality (50 of 179 vs. 67 of 277; heterogeneity *p* = .688; *I* ^2^ = .0%; OR = .966, 95% CI: .614–1.518; *p* = .880; Figure 4). Of note, patients undergoing first‐line aspiration had a significantly lower rate of postoperative hemorrhagic complication than patients undergoing stent retriever (26 of 218 vs. 66 of 404; heterogeneity *p* = .242; *I* ^2^ = 25.6%; OR = .446, 95% CI: .259–.769; *p* = .004; Figure 5). Furthermore, we performed a subgroup analysis on subarachnoid and parenchymal hematoma, no significant difference was observed on postoperative subarachnoid (0 of 83 vs. 11 of 183; heterogeneity *p* = .801; *I* ^2^ = .0%; OR = .171, 95% CI: .022–1.349; *p* = .094; Figure 5) and parenchymal hematoma (4 of 106 vs. 9 of 195; heterogeneity *p* = .561; *I* ^2^ = .0%; OR = .799, 95% CI: .236–2.712; *p* = .720; Figure 5).

### 3.4 Other outcomes of interest

#### 3.4.1 Favorable outcome

**FIGURE 6:**
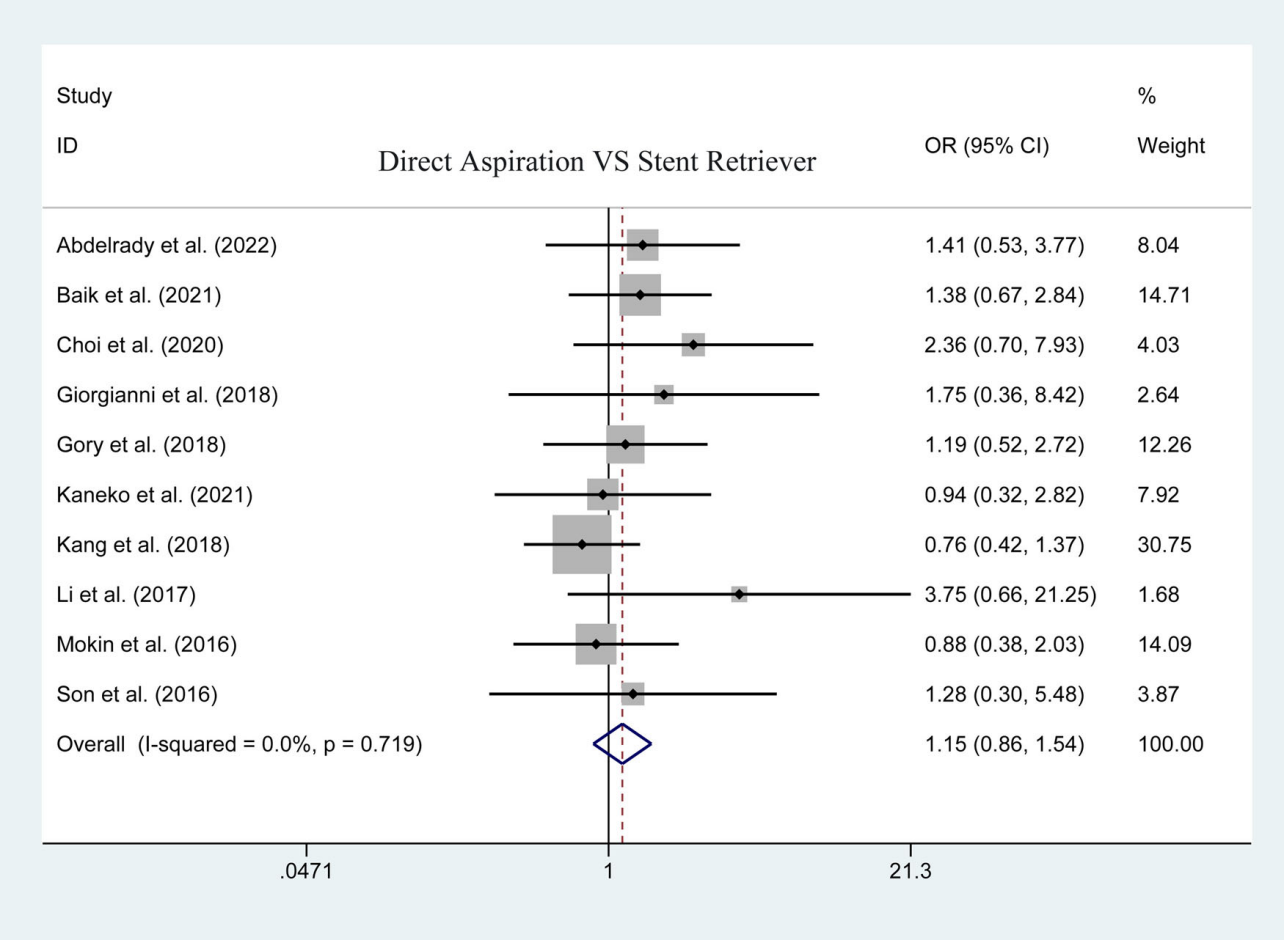
Forest plot for assessing postoperative favorable outcome.

A comparison of postoperative favorable outcomes was reported by 10 of the included studies. The pooled results indicated that there was no significant difference between the two groups (123 of 319 vs. 213 of 601; heterogeneity *p* = .719; *I* ^2^ = .0%; OR = 1.149, 95% CI: .858–1.540; *p* = .352; Figure 6).

#### 3.4.2 Rescue therapy

**FIGURE 7:**
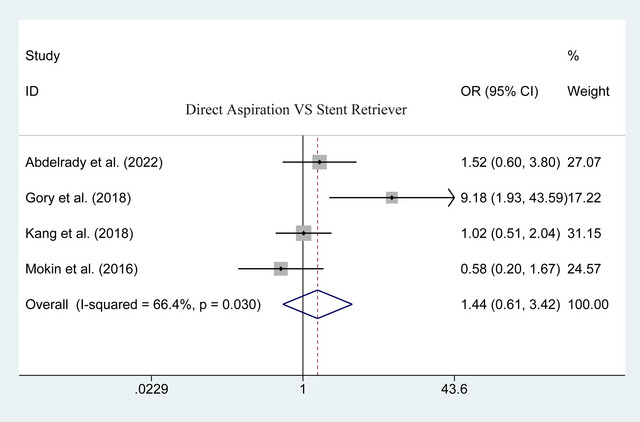
Forest plot for assessing postoperative rescue therapy.

A comparison of postoperative rescue therapy was reported by 4 of the included studies. The pooled results indicated that there was no significant difference between the two groups (53 of 208 vs. 57 of 292; heterogeneity *p* = .030; *I* ^2^ = 66.4%; OR = 1.440, 95% CI: .606–3.421; *p* = .409; Figure 7).

#### 3.4.3 Procedure duration

**FIGURE 8:**
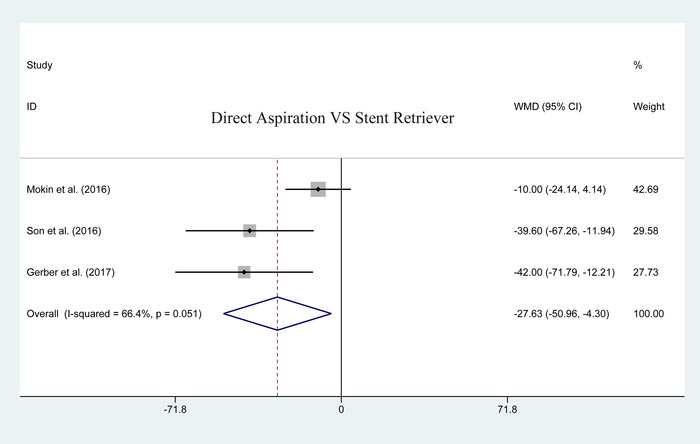
Forest plot for assessing postoperative procedure duration.

Procedure duration was a continuous variable, a total of three studies reported data on the outcome of procedure duration. It was represented as a WMD with 95% CIs. The pooled results revealed a significant difference between the two groups in favor of aspiration (heterogeneity *p* = .051; *I* ^2^ = 66.4%; WMD = −27.630, 95% CI: −50.958 to −4.302; *p* = .020; Figure 8). More details are shown in Table 3.

**TABLE 3:** The outcomes of this meta‐analysis.

		Sample size		Overall effect			Heterogeneity	
Outcomes	Studies numbers	Direct aspiration	Stent retriever	Effect estimates	95% CIs	*p*‐Value	*I* ^2^ (%)	*p*‐Value
The results concerning post‐operation recanalization of aspiration vs. stent retriever								
Successful recanalization	9	271/312	429/530	OR = 1.642	1.099–2.453	***p* = .015**	.0	*p* = .892
Complete recanalization	4	111/176	172/330	OR = 1.936	1.306–2.872	***p* = .001**	19.8	*p* = .291
The results concerning post‐operation complication of aspiration vs. stent retriever								
Total complication	5	92/205	202/389	OR = .359	.229–.563	***p* < .001**	47.5	*p* = .106
Hemorrhagic complication	6	26//218	66/404	OR = .446	.259–.769	***p* = .004**	25.6	*p* = .242
Mortality	5	50/179	67/277	OR = .966	.614–1.518	*p* = .880	.0	*p* = .688
Subarachnoid hematoma	2	0/83	11/183	OR = .171	.022–1.349	*p* = .094	.0	*p* = .801
Parenchymal hematoma	2	4/106	9/195	OR = .799	.236–2.712	*p* = .720	.0	*p* = .561
The results concerning the others of interest of aspiration vs. stent retriever								
Favorable outcome	10	123/319	213/601	OR = 1.149	.858–1.540	*p* = .352	.0	*p* = .719
Rescue therapy	4	53/208	57/292	OR = 1.440	.606–3.421	*p* = .409	66.4	.030
Procedure duration	3	80	84	WMD = −27.630	−50.958 to −4.302	***p* = .020**	66.4	*p* = .051

Abbreviations: CIs, confidence intervals; OR, odds ratio; WMD, weighted mean difference.

The bold means P<0.05.

## 4 DISCUSSION

Basilar artery occlusion is a rare kind of acute stroke; clinical outcomes vary, but the condition can be fatal (Alemseged et al., 2023; Mortimer et al., 2012). Due to the fact that patients with basilar artery occlusion were typically not included in trials of endovascular thrombectomy for large‐vessel occlusion ischemic stroke, the efficiency of this treatment was unknown until recently (Alemseged et al., 2023). Previous studies indicated that recanalized patients experience significantly better results than unrecanalized patients, and it was well acknowledged that endovascular thrombectomy provides high rates of recanalization (Alemseged et al., 2023). Importantly, numerous studies have demonstrated that in patients with basilar artery occlusion, stent retrievers might also produce a high rate of recanalization and functional independence (Gory et al., 2016; Phan et al., 2016). A first‐line aspiration, according to many researchers, has advanced quickly, demonstrated efficacy and safety for recanalization for acute ischemic stroke (Vargas et al., 2017), and it may be superior to stent retrievers (Lapergue et al., 2016). This study aimed to compare the stent retriever and direct aspiration concerning reported recanalization rates and periprocedural complications. In this intention‐to‐treat meta‐analysis of the retrospective and prospective cohort study, our findings substantiated that first‐line aspiration, as compared to stent retriever, led to a notable enhancement in recanalization rates, accompanied by a significant reduction in both procedural complications and duration in patients with acute basilar artery occlusion.

Before the evolution in endovascular thrombectomy devices, three small single‐center retrospective studies evaluated the postoperative revascularization of aspiration and stent retriever in basilar artery occlusion in 31 (18 in aspiration vs. 13 in stent retriever), 33 (20 in aspiration vs. 13 in stent retriever), and 50 (16 in aspiration vs. 34 in stent retriever) patients (Choi et al., 2020; Gerber et al., 2017; Son et al., 2016). A significant discrepancy in the two groups was found with reference to postoperative revascularization. In 100 patients with basilar artery obstruction, Gory et al. (2018) further verified these findings and found that aspiration was related to noticeably greater odds (2.6‐fold) of complete revascularization. In contrast, the level of complete revascularization did not differ between the two devices in a previous meta‐analysis, but the aspiration group had a higher rate of successful revascularization (Ye et al., 2019). In this study, we identified a significant difference in successful recanalization (OR = 1.642; 95% CI: 1.099–2.453; *p* = .015) and complete recanalization (OR = 1.936; 95% CI: 1.306–2.872; *p* = .001) between the two groups in favor of the first‐line aspiration. Importantly, our pooled results suggested that the first‐line aspiration technique achieves a faster recanalization for patients with acute basilar artery occlusion compared with the stent retriever (WMD = −27.630, 95% CI: −50.958 to −4.302; *p* = .020). Aspiration is theoretically simpler than stent retrieval because the aspiration catheter does not need to pass through the blood clot to reach the proximal end of the thrombus. However, it is important to note that this situation is not universally applicable. There are instances where reaching the thrombus solely with the aspiration catheter proves challenging, particularly in elderly patients with elongated vessels. In such cases, the support of a microwire or microcatheter becomes essential. This reliance on additional devices also accounts for the occurrence of a nonzero perforation rate in aspiration procedures. Besides that, no significant discrepancies were found with reference to rescue therapy (53 of 208 vs. 57 of 292; OR = 1.440, 95% CI: .606–3.421; *p* = .409); therefore, there is no additional time for preparing another pass or for switching to other rescue therapies.

Our data also supported the notion that aspiration therapy was a safer approach than stent retrieval. Aspiration treatment was recommended in each of the five studies that showed complications after surgery. None of the other four investigations, however, demonstrated a statistically significant difference in complications between the two groups, with the exception of the study by (Abdelrady et al., 2023) The combined data revealed that individuals who underwent aspiration had a lower incidence of total complications than those who underwent stent retrieval (OR = .359; 95% CI: .229–.563; *p* < .001). Of note, a higher incidence of postoperative mortality was not detected in the aspiration group (50 of 179 vs. 67 of 277; OR = .966, 95% CI: .614–1.518; *p* = .880). In addition, the combined findings showed that the aspiration group saw fewer hemorrhagic consequences (26 of 218 vs. 66 of 404; OR = .446, 95% CI: .259–.769; *p* = .004). Upon the specific hemorrhagic complications, however, no significant difference was observed on postoperative subarachnoid (0 of 83 vs. 11 of 183; OR = .171, 95% CI: .022–1.349; *p* = .094) and parenchymal hematoma (4 of 106 vs. 9 of 195; OR = .799, 95% CI: .236–2.712; *p* = .720). However, it is important to note that there was a trend in favor of aspiration, indicating that vascular perforation may result from blind wire passage. The basilar artery is more susceptible to this possibility than the anterior circulation, which is more susceptible to hemorrhagic consequences (Choi et al., 2020).

Although thromboembolism is a more common cause of ischemic stroke with large and medium size vessel occlusion in the anterior circulation, the underlying etiology of basilar artery occlusion is often associated with cardioembolism, large artery atherosclerosis, and other mechanisms such as dissection and vasculopathy (Buchman & Merkler, 2019). Importantly, basilar artery occlusion stemming from atherosclerotic disease tends to be linked with unfavorable clinical outcomes (Lee et al., 2017; Mutke et al., 2023). During a basilar artery occlusion, atherosclerotic occlusions are commonly observed in proximal locations, whereas thromboembolic occlusions tend to occur more frequently in distal locations (Mutke et al., 2023). Patients diagnosed with atherosclerotic or embolic basilar artery occlusion may necessitate distinct treatment approaches and might experience varying benefits from the best endovascular stroke therapy. In addition, first‐line approaches will probably be tailored to the specific etiology of stroke, and it is possible that aspiration thrombectomy may not be suitable or could potentially pose risks for patients with atherosclerotic vascular disease.

To the best of my knowledge, this is the second meta‐analysis assessing the efficacy and safety of the stent retriever and direct aspiration with regard to reported recanalization rates and periprocedural complications. Ye et al. (2019) included 5 cohort studies (2 prospective and 3 retrospective) and 476 cases, they indicated a similar observation with our study. It is clear that this study included more studies with larger numbers of patients. The following limitations of this study should be noted. The included studies’ participant characteristics and primary inclusion and exclusion criteria for trials differ, potentially leading to bias. There was some heterogeneity across the included trials in terms of study methods, patient characteristics, and clinical outcome criteria. Meanwhile, for the subgroup analysis, we could not divide the participants into groups with different follow‐up times due to relatively little reported data. Although we included 11 studies (3 prospective and 8 retrospective studies), none of them were randomized controlled trials. It is necessary to conduct additional randomized controlled trials with larger sample numbers to verify this conclusion.

## 5 CONCLUSION

Endovascular treatment using the first‐line aspiration was associated with a higher rate of recanalization, a lower risk of postoperative complication, and a shorter duration of the procedure. Based on this evidence, these findings support the aspiration may be more secure than a stent retriever. However, given that the limitations arose from this analysis, more evidence‐based performance is needed to supplement this opinion.

## AUTHOR CONTRIBUTIONS

Yanmei Ju and Hongxin Jiang designed and conceptualized the study. Yongbin Wang and Juan Zhang finished data extraction from literature search. It was Juan Zhang who created the tables and figures. Each contributor made a significant contribution to the paper's composition as well as providing valuable intellectual insight.

## CONFLICT OF INTEREST STATEMENT

The authors report no conflicts of interest.

### PEER REVIEW

The peer review history for this article is available at https://publons.com/publon/10.1002/brb3.3141.

## Data Availability

The data that support the findings of this study are available from the corresponding authors upon reasonable request.
